# Comprehensive analysis of PTPN gene family revealing PTPN7 as a novel biomarker for immuno-hot tumors in breast cancer

**DOI:** 10.3389/fgene.2022.981603

**Published:** 2022-09-26

**Authors:** Fengxu Wang, Xuehai Wang, Lei Liu, Siyuan Deng, Wenqian Ji, Yang Liu, Xiangdong Wang, Rui Wang, Xinyuan Zhao, Erli Gao

**Affiliations:** ^1^ Department of Occupational Medicine and Environmental Toxicology, Nantong Key Laboratory of Environmental Toxicology, School of Public Health, Nantong University, Nantong, China; ^2^ Department of Pathology, Affiliated Hospital of Nantong University, Nantong, China; ^3^ College of International Studies, Southwest University, Chongqing, China; ^4^ Department of General Surgery, The First Affiliated Hospital of Soochow University, Suzhou, China

**Keywords:** PTPN, bioinformatics, breast cancer, immune infiltration, biomarker

## Abstract

**Background:** The non-receptor protein tyrosine phosphatase (PTPN) gene family has been considered to be involved in the oncogenesis and development of multiple cancers. However, its prognostic utility and immunological relevance in breast cancer (BrCa) have not been clarified.

**Methods:** A transcriptional level interpretation of the expressions and prognostic values was analyzed using the data from The Cancer Genome Atlas (TCGA) cohort. In addition, GO and DAVID pinpoint the functional enrichment of PTPNs. Moreover, the immune correlations of PTPN7 in BrCa and pan-cancer were further investigated based on the TCGA cohort and were testified using the in-house and the Gene Expression Omnibus (GEO) cohorts.

**Results:** For systematic analysis of the PTPN family, we found that the expression levels of PTPN1, PTPN6, PTPN7, PTPN18, PTPN20, and PTPN22 was promoted in tumor tissues while comparing with paraneoplastic tissues during our study. We further investigated their functions and protein-protein interactions (PPI), and these results strongly suggested that PTPN family was associated with protein dephosphorylation. Next, we performed an immunological relevance analysis and found that PTPN7 was correlated with immune infiltration, suggesting a stronger association of PTPN7 with immuno-hot tumors in BrCa. In addition, results from the in-house cohort confirmed the positive correlation between PTPN7 and PD-L1. The pan-cancer analysis revealed that PTPN7 was related to PD-L1 and CTLA-4 expression in almost all cancer types. Finally, the predictive value of PTPN7 for immunotherapy was significant in two independent GEO cohorts.

**Conclusion:** In conclusion, this is the first extensive research on the correlation between PTPN family expression and immune characterization in BrCa. As results, PTPN7 expression is associated with immuno-hot tumors and could be a promising predictive biomarker for immunotherapy in not only BrCa but multiple cancers.

## 1 Introduction

Breast cancer (BrCa) is one of the most frequent kinds of cancer and is the common cause of cancer-related death among female worldwide (Wang et al., 2021a). There will be around 51,400 new cases of BrCa in 2022, which ranks the first among all cancers (Siegel et al., 2022). The morbidity and mortality of BrCa vary from country to country, with age-standardized incidence ranging from a high of 112.3 per 100,000 in Belgium to a low of 35.8 per 100,000 (Lei et al., 2021). Early detection and diagnosis of BrCa are crucial to improving curative effects and clinical outcomes. Treatments for BrCa includes surgical excision, radiotherapy, endocrine therapy, chemotherapy, and immunotherapy (Trayes and Cokenakes, 2021). Tumor biomarkers are representatives of these characteristics used to predict outcomes. What’s more, there are various outcomes while the immunotherapy involved in BrCa caused by individual presentation. Unpredictable, unrealizable, and uncountable situation happened in BrCa immunotherapy. (Emens, 2018) Additionally, due to the lack of BrCa-specific biomarker the reaction for different subtypes of BrCa is intricate and unexplainable such as the TNBC is an immune-hot type cancer for consider. (Howlader et al., 2018) However, the incomprehensive recognition of BrCa take the challenge of BrCa treatment. Therefore, it is necessary to identify novel biomarkers that are meaningful for the prognostic evaluation and therapeutic prediction in BrCa.

Non-receptor protein tyrosine phosphatase (PTPN) is a group of enzymes involved in tyrosine phosphorylation (Chen et al., 2020). Tyrosyl phosphorylation is a bidirectional and elastic process that plays a key role in many cellular signaling pathways (Li et al., 2013). PTPNs are encoded by 103 genes divided into four major superfamily categories, each of which is given a formal gene name by the Human Genome Organization in the Nomenclature Committee (Alonso et al., 2004). The 17 non-receptor PTPs belonging to the biggest family class I are labeled as PTPNs, followed by numbers, under the latter scheme. More evidence has emerged in recent years indicating members of the PTPN gene family are involved in a wide range of physiological and pathological processes, including cell proliferation, immunological response, and metabolism (Lu et al., 2022). Similarly, members of the PTPN family have a role in the oncogenesis and development of multiple malignancies. For example, PTPN12 plays an essential role in tumor growth and the transformation of triple-negative breast cancer (Thummuri et al., 2015; Nair et al., 2018). Simultaneously, PTPN3 inhibits the growth of lung neuroendocrine cancer (Koga et al., 2021). Moreover, PTPN18 inhibits the metastasis of endometrial cancer cells (Cai et al., 2019). In general, this evidence makes the PTPNs promising prognostic and therapeutic targets for cancer therapy. However, the unique functions and immuno-correlations of PTPN family genes in BrCa have not been fully elucidated so far.

In this study, we investigated the expression of PTPN in BrCa specimens and discussed its clinical characteristics using The Cancer Genome Atlas (TCGA) database. We also comprehensively analyzed the correlation between PTPNs and characteristics of tumor microenvironment (TME). In addition, an in-house cohort was used to confirm the correlation between PTPN7 and PD-L1 at the protein level in BrCa. Moreover, the pan-cancer analysis of PTPN7 was also conducted, which revealed that PTPN7 might be a novel predictive biomarker for immunotherapy.

## 2 Materials and methods

### 2.1 Public data acquisition

The data of RNA-sequencing (RNA-seq) and clinical information in the TCGA were downloaded from the UCSC Xena data portal (https://xenabrowser.net/). In addition, the GSE35640 (Ulloa-Montoya et al., 2013), GSE126044 are the non-small cell lung cancer datasets. Moreover, GSE35640 (Ulloa-Montoya et al., 2013)is early-stage lung cancer dataset and PRJNA558949 (Blenman et al., 2022) is the TNBC dataset. Except PRJNA558949 is from the NCIB BioProject, the others are from the GEO database. All of datasets are the RNA-seq data from cancer patients receiving immunotherapy, were also acquired from the official website (https://www.ncbi.nlm.nih.gov/geo/).

### 2.2 Kaplan-meier plotter database analysis

Kaplan-Meier plotter (https://kmplot.com/analysis/) is a website’s tool comprising gene expression cohorts, clinical information, survival data which contained multiple types of cancer patients (Sun et al., 2019). All cancer samples accessible on the Kaplan-Meier plotter were aimed to evaluate the prognostic values of PTPNs in BrCa. The patients, who is diagnosed with BrCa, were divided into cohorts based on expression levels of PTPNs in the median reproduction, with the rest of the settings set to default. Kaplan-Meier survival plots were derived into display all of the cohorts. The log-rank *p*-value, 95 percent confidence interval (95%CI), and hazard ratio (HR) were computed and shown online.

### 2.3 Database for annotation, visualization, and integrated discovery database analysis

The Database for Annotation, Visualization, and Integrated Discovery (DAVID) was employed to perform gene ontology (GO) analysis of PTPNs (Dennis et al., 2003). The background variable was chosen to be the human genome (*Homo sapiens*). When the false discovery rate (FDR) was less than 0.05, enrichment terms were judged statistically significant.

### 2.4 Protein-protein interaction network construction

GeneMANIA is a dynamic and visual protein-protein interaction (PPI) prediction tool based on website, which aimed to create a customizable function for the inspection of genes with comparable functions (Warde-Farley et al., 2010; Esmaeili et al., 2019). GeneMANIA was used to analyze the PPI of PTPN family members in this study. The STRING database incorporates data from a variety of sources, including experimental data, computer prediction approaches, and publicly available text collections. It is open to the public and is updated on a regular basis (Szklarczyk et al., 2021). In this research, we used the STRING tool to construct the PPI network of PTPNs and the result was visualized with Cytoscape. The MCC algorithm was used to identify the PPI hub gene in the network.

### 2.5 Estimation of the immunological characteristics of the TME

The tumor Immune Estimation Resource (TIMER) database is an online tool for systematic analysis of immune cell infiltration across diverse cancer types from TCGA (Li et al., 2017). TIMER uses a deconvolution algorithm to estimate the abundance of tumor-infiltrating immune cells (TIICs) based on gene expression profiles (Dong et al., 2020). We evaluated PTPN7 and PTPN22 expression in multiple cancers and the correlation of PTPN7 and PTPN22 expression with the abundance of TIICs, including B cells, CD8^+^ T cells, and CD4^+^ T cells, neutrophils, macrophages, and dendritic cells, and the tumor purity. To validate the involvement of PTPN7 in cancer immunity in BrCa, we assessed the correlations between PTPN7 and immune checkpoints expression, immune cell markers expression, as well as IPS. In addition, we split the patients into high- and low-PTPN7 groups based on PTPN7 transcriptional levels using a 50 percent threshold and compared tumor mutation burden (TMB) in BrCa.

### 2.6 Linked omics database analysis

A web-based tool for evaluating multidimensional data cohorts is the Linked Omics database (http://www.linkedomics.org/login.php) (Vasaikar et al., 2018). Gene set enrichment analysis was used to predict the functional functions of PTPN7 in BrCa using the Linked Omics tool in the term of Gene Ontology (GO) analysis. For all parameters, the default choices were utilized.

### 2.7 Clinical samples

The BrCa tissue microarray (TMA, Cat. HBre-Duc060CS-03) was obtained from Outdo BioTech (Shanghai, China). The tissue microarray contained 30 tumor samples and 30 paired adjacent samples. Detailed clinicopathological characteristics of the cohorts were provided by Outdo BioTech. The tissue microarray was submitted for immunohistochemistry (IHC) staining in this research. Ethical approval (YB-M-05–02) for the study of tissue microarray slides was granted by the Clinical Research Ethics Committee, Outdo Biotech (Shanghai, China).

### 2.8 Immunohistochemistry staining

Immunohistochemistry (IHC) staining was directly conducted on the HBre-Duc060CS-03 TMA with standard procedures. The primary antibodies used were as follows: anti-PTPN7 (1: 800 dilution, 15286-1-AP, Proteintech, Wuhan, China) and anti-PD-L1 (Ready-to-use, Cat. GT2280, GeneTech, Shanghai, China). Antibody staining was visualized with DAB and hematoxylin staining, and stained sections were scanned using Aperio Digital Pathology Slide Scanners. The stained sections were examined separately by two pathologists according to the assessment criteria on a 12-point scale by generating the immunoreactivity score (IRS) for semi-quantitative analysis. The percentage of positively stained cells was scored as 0–4: 0 (<5%), 1 (6–25%), 2 (26–50%), 3 (51–75%) and 4 (>75%). The staining intensity was scored as 0–3: 0 (negative), 1 (weak), 2 (moderate), and 3 (strong). The immunoreactivity score (IRS) equals to the percentages of positive cells multiplied with staining intensity (Mei et al., 2021).

### 2.9 Statistical analysis

R 4.0.2 and Graphpad Prism 6 were used to conduct all of the statistical studies depicted in the figures. To see if there was an obvious difference in continuous parameters between the two groups. The Wilcoxon rank-sum test was utilized which aimed for comparing categorical variables, the chi-square test was performed. The log-rank test was used to analyze the prognostic significance of categorical variables. If not stated otherwise, a two-paired *p*-value is no more than 0.05 that deemed statistically tremendous in all analyses. *p < value 0.05, **p < value 0.01, ***p < value 0.001, and ****p < value 0.0001 were used to determine statistical significance.

## 3 Results

### 3.1 Expression levels, mutations, and prognostic values of PTPNs in BRCA

**FIGURE 1:**
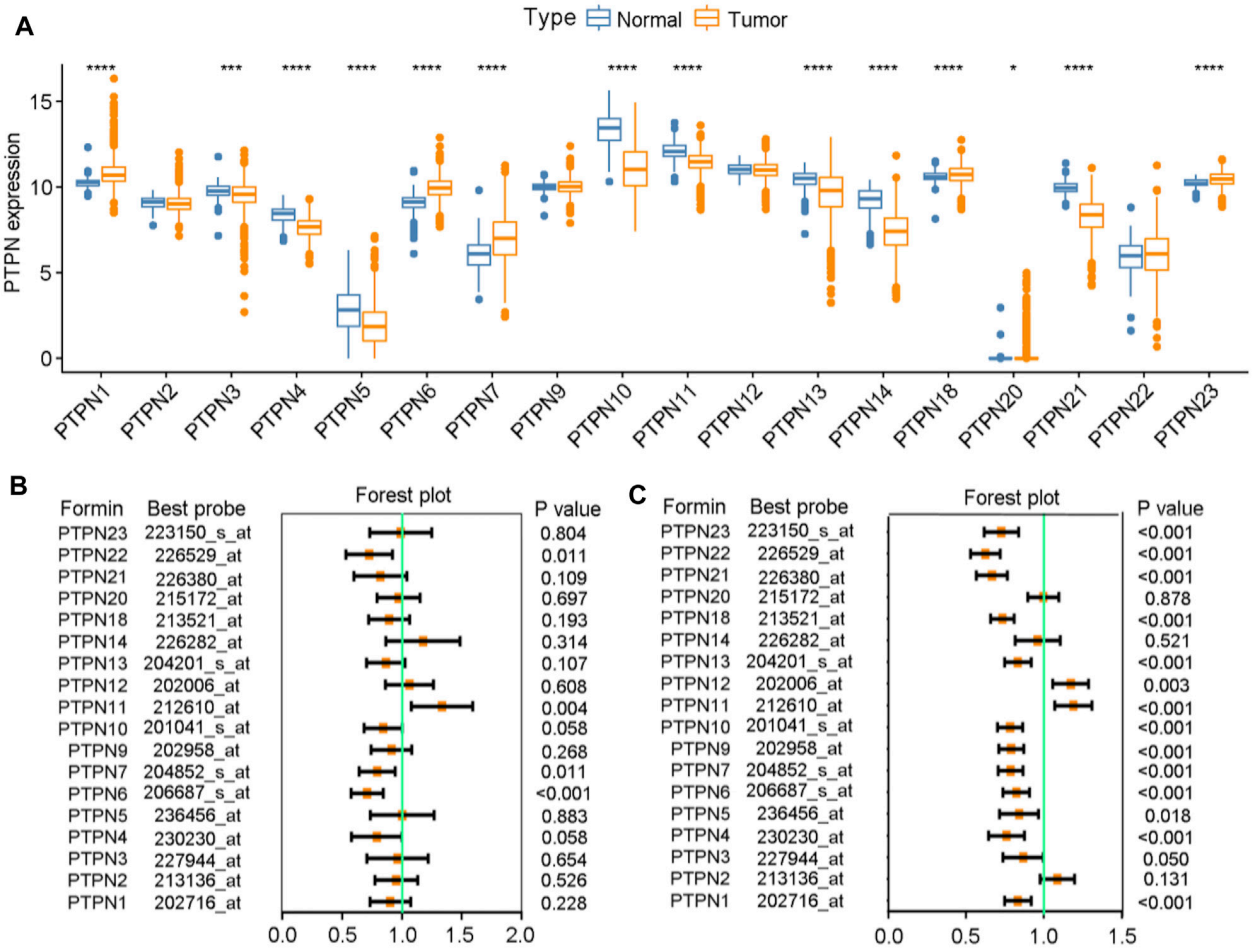
Expression levels and prognostic values of PTPNs in BrCa. **(A)** Expression levels of PTPNs genes in the normal and tumor groups. **(B)** Forest plot displaying the prognostic values of PTPNs for OS generated by using the Kaplan-Meier plotter database. **(C)** Forest plot displaying the prognostic values of PTPNs for RFS generated by using the Kaplan-Meier plotter database.

Initially, a thorough examination of the expressions as well as prognostic significance of PTPNs in BrCa was carried out. We analyzed the expression levels of PTPNs in the TCGA database and discovered that PTPN1, PTPN6, PTPN7, PTPN18, PTPN20, and PTPN22 were significantly upregulated in tumor samples, while PTPN4, PTPN5, PTPN10, PTPN11, PTPN13, PTPN14, and PTPN21 were downregulated in tumor samples (Figure 1A). In addition, we also analyzed the mutation of PTPNs and found that the mutation rates of PTPTNs were overall low (Supplementary Figure S1). Based on the analysis of patients’ overall survival (OS) and relapse-free survival (RFS), we found that some members of the PTPN family may be associated with a better prognosis in BrCa. Regarding OS, with the downregulation of PTPN1/4/6/7/10/13/18/21/21, the worse prognosis was established, while with the downregulation of PTPN11/14, the better prognosis happened (Figure 1B). Regarding RFS, we observed that most of PTPNs expression were associated with worse prognosis, except PTPN2/11/12 (Figure 1C). Through the above preliminary analysis, we can conclude that the PTPN family has the value of further research in BrCa.

### 3.2 Functions and PPI construction of PTPNs

**FIGURE 2:**
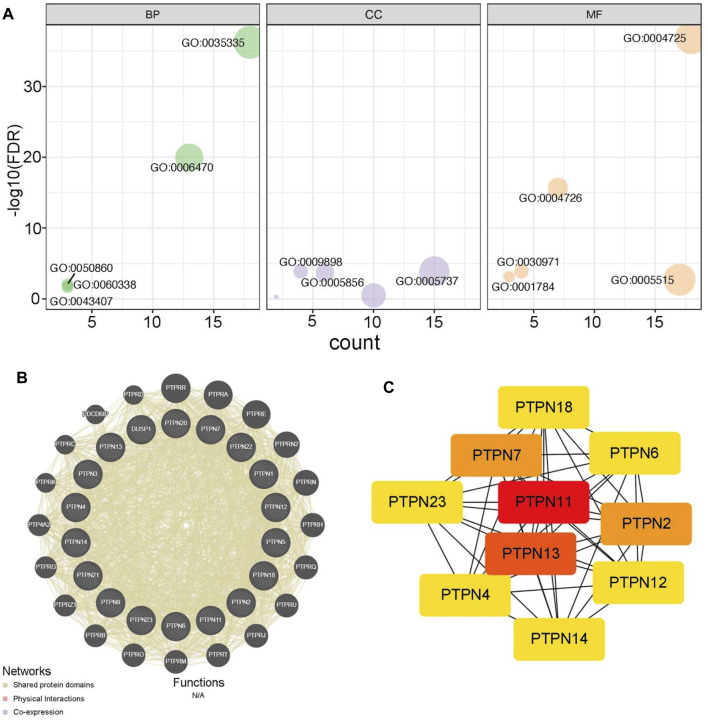
Functions of PTPNs and their interacted networks. **(A)** GO analysis of PTPN family based on the DAVID tool. **(B)** Predicted pathways of PTPNs using the GeneMANIA tool. The interconnections between proteins were explored in terms of physical interaction, coexpression and shared protein domains. **(C)** PPI network of PTPNs constructed using the STRING tool and visualized using the Cytoscape tool.

Next, we analyzed the potential functions of PTPNs. As showed in Figure 2A, PTPNs mainly participated in peptidyl-tyrosine dephosphorylation, located in cytoplasm, and regulated protein tyrosine phosphatase activity (Figure 2A, Supplementary Table S1). In addition, we exhibited the PPI network of PTPNs and their partners using the GeneMANIA tool (Figure 2B). Moreover, we identified PTPN11 as the central member of PTPNs using the STRING and Cytoscape tools (Figure 2C). Overall, these results provide preliminary insight into the functions of PTPNs.

### 3.3 Immunological correlations of PTPNs in BrCa

**FIGURE 3:**
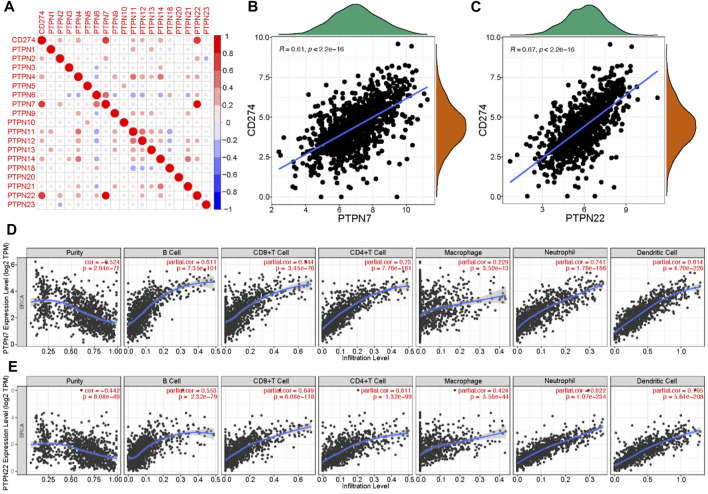
Correlations between PTPNs and PD-L1 as well as immune infiltration in BrCa. **(A)** PTPNs and their correlation with programmed cell death-ligand 1. Red: positive association, blue: negative association. **(B,C)** Correlation between PTPN7 and PTPN22 and PD-L1. **(D,E)** Correlation of PTPNs expression with immune infiltrating level in BrCa. expressions were significantly positively related to tumor purity. While PTPN7 and PTPN22 expressions were significantly positively correlated with infiltrating levels of CD8 T cells, CD4 T cells, macrophages, neutrophils, and dendritic cells in BrCa.

Malignant tumor cells utilize immunosuppressive means to against anti-tumor immunity. The approach to interrupt the PD-1/PD-L1 pathway is named as immune checkpoint blockade (Hamanishi and Konishi, 2014). Given the significant role of PD-L1 in tumor immunity, we next evaluated the correlations between PTPNs and PD-L1 expression. The expression of several PTPNs was positively correlated with PD-L1 (Figure 3A), among which PTPN7 and PTPN22 had the strongest correlation with PD-L1 (Figures 3B,C). An abundance of TIICs in cancer has been utilized to forecast cancer sentinel lymph node suasion and prognosis (Bodurtha Smith et al., 2017). High level mRNA expressions of PTPN7 and PTPN22 were significantly negatively correlated with tumor purity. In addition, the expression of PTPN7 and PTPN22 mRNA was optimistically correlated with the infiltration levels of B cells, CD8^+^ T cells, and CD4^+^ T cells, neutrophils, macrophages, and dendritic cells. These results strongly suggest that PTPNs are contained promising relationship with immune infiltration in BrCa (Figures 3E,F).

### 3.4 PTPN7 predicts inflamed TME in BrCa

**FIGURE 4:**
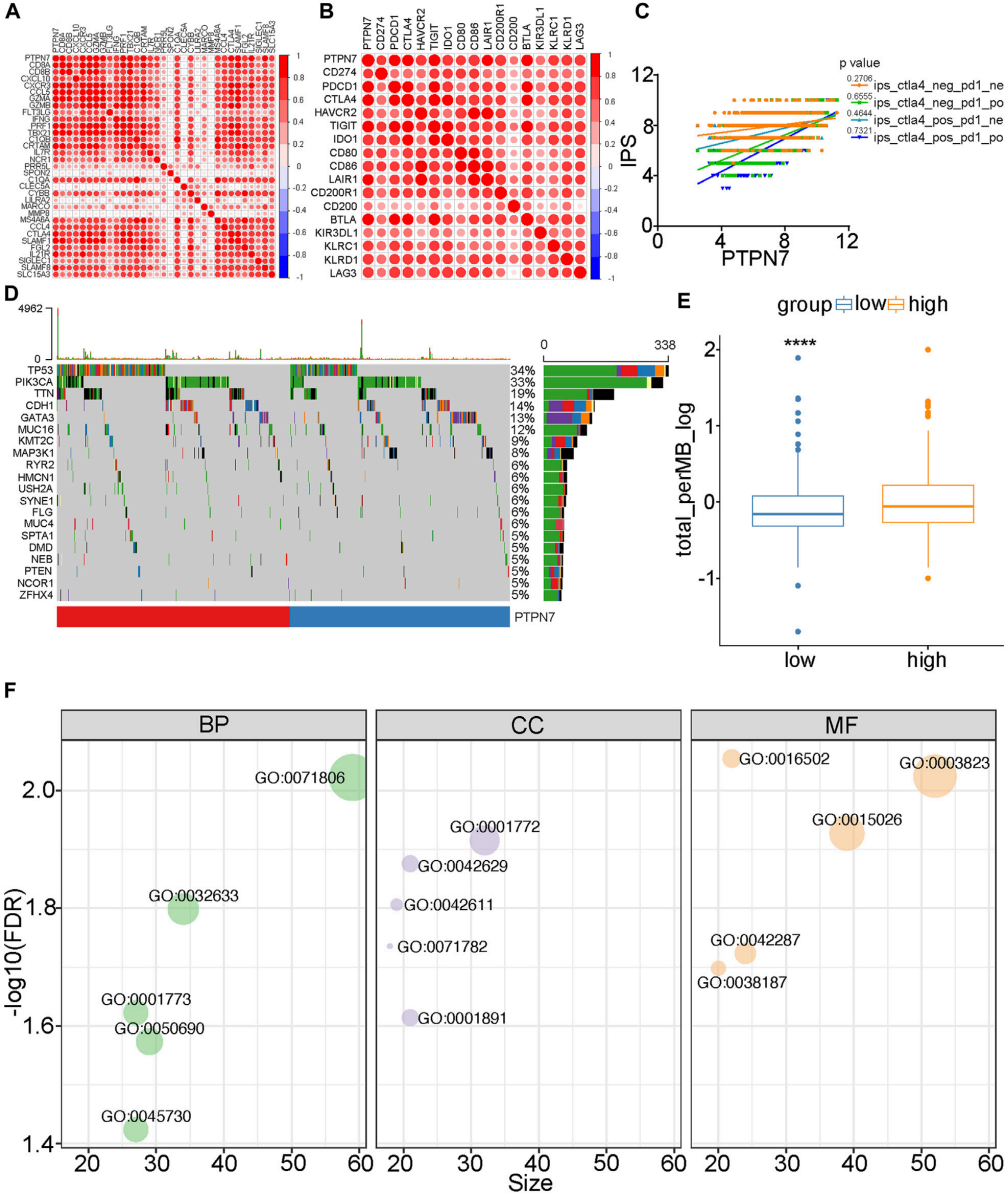
Immuno-correlations between PTPN7 in BrCa and its functions. **(A)** Correlations between PTPN7 and gene markers for immune cells. **(B)** Correlations between PTPN7 and common inhibitory immune checkpoints. **(C)** The different PTPN7 expression that the IPS are existing in four types which is pd1&ctla4 positive or negative. **(D)** Mutational profiles of commone genes in the different PTPN7 expression group in the TCGA cohort. **(E)** Comparison of PTPN7 expression in high and low PTPN7 groups. **(F)** GO analysis of PTPN7 in BrCa based on the Linked Omics tool.

**FIGURE 5:**
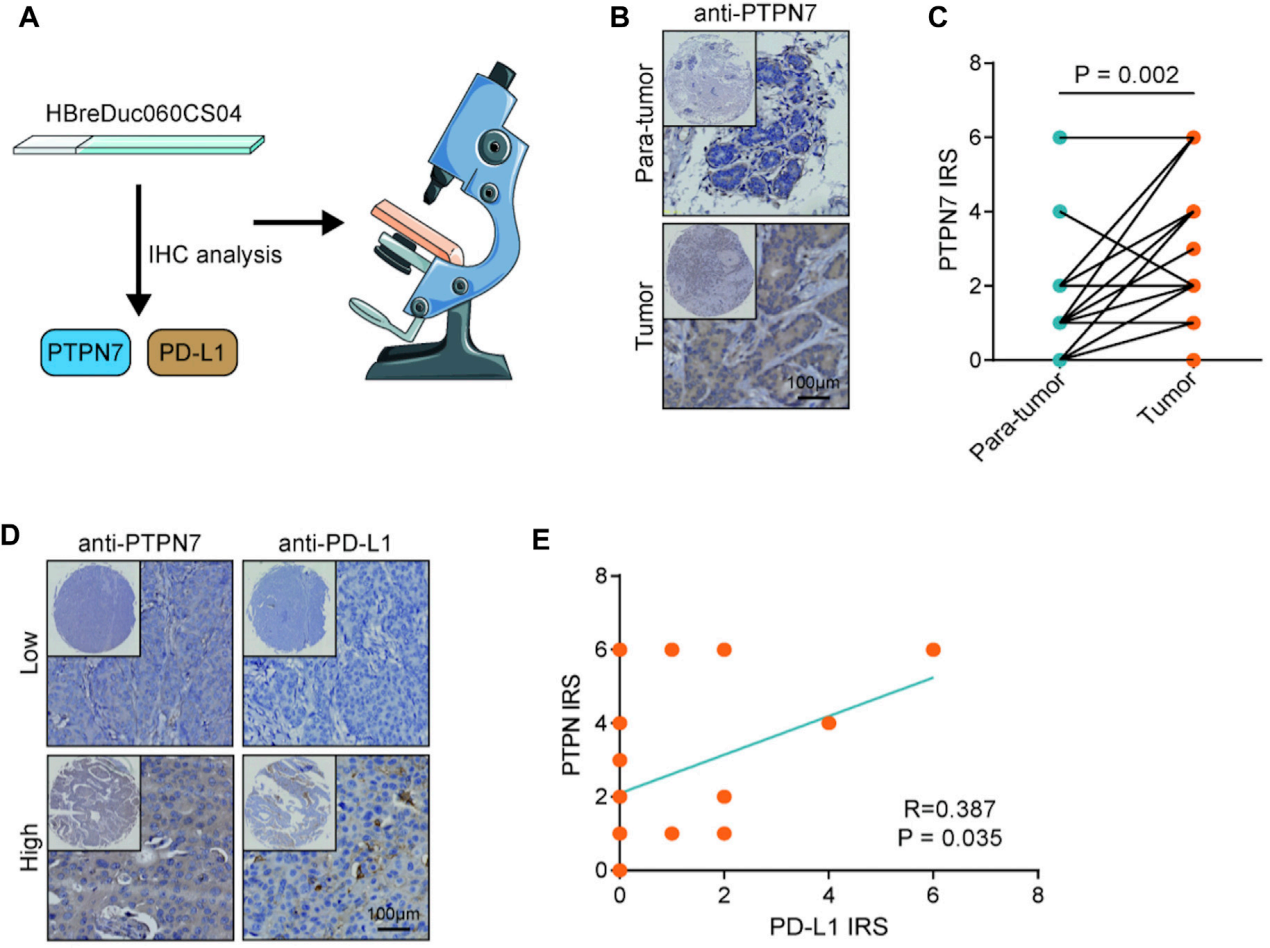
Validation of PTPN7 expression in BrCa and its correlation with PD-L1. **(A)** Using the TMA which contained PTPN7 & PD-L1 and acquiring the tissue images. **(B)** Representative images revealing PTPN7 expression in tumor and para-tumor tissues. Magnification, 200×. **(C)** Expression levels of PTPN7 in tumor and para-tumor tissues. **(D)** Representative images revealing PD-L1 expression in the high and low PTPN7 groups. Magnification, 200× **(E)** Correlation between PTPN7 and PD-L1 expression.

Both PTPN7 and 22 were correlated with immune invasion, but the high expression of PTPN7 was associated with a better prognosis, suggesting that PTPN7 was more correlated with immuno-hot tumors. Because previous studies indicated that immuno-hot tumors have a better prognosis (Cai et al., 2021). Thus, we next performed an in-depth analysis of PTPN7 in BrCa. First, PTPN7 was positively correlated with most gene markers of multiple immune cells (Figure 4A). In addition, PTPN7 was also positively correlated with multiple immune checkpoints expression (Figure 4B). Moreover, one of our interpretations revealed that PTPN7 was affirmatively correlated with IPS (Figure 4C). Additionally, TMB and mutation rates of several critical genes, including TP53, PIK3CA, CDH1, TTN, GATA3, and KMT2C were tremendously increased in the high PTPN7 cohort (Figures 4D,E). Then, the GO interpretation based on Linked Omics were driven to embellish the functional enrichment of PTPN7 and their genes (Figure 4F, Supplementary Table S2). Next, HBreDuc060CS04 TMA was used as a validation cohort (Figure 5A). In the term of PTPN7 expression, there was a substantial difference between BrCa and paracancerous tissues which revealed the PTPN7 expression was higher in tumor tissues (Figures 5B,C). In addition, we found that PTPN7 was positively correlated with PD-L1 expression in the current cohort (Figures 5D,E). Overall, these results reveal that PTPN7 is related to immuno-hot tumors in BrCa.

### 3.5 Immunological correlation of PTPN7 in pan-cancer

**FIGURE 6:**
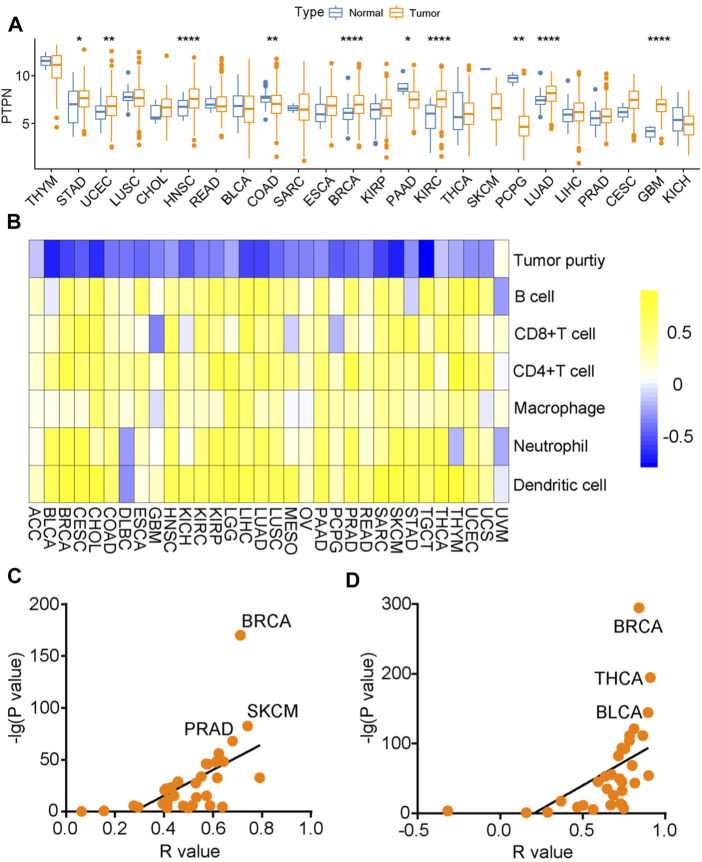
Immuno-correlation of PTPN7 in pan-cancer. **(A)** Expression of PTPN7 in pan-cancer. **(B)** Correlations between PTPN7 and 6 TIICs calculated with the TIMER algorithm. The color indicates the correlation coefficient. **(C,D)** The correlation between PTPN7 and PD-L1 &CTLA-4 in various tumor types.

Subsequently, we analyzed the expression of PTPN7 and its immunological correlation in pan-cancer tissues. Compared with para-tumor specimens, PTPN7 was upregulated in tumor specimens of STAD, CHOL, HNSC, ESCA, BRCA, KIPR, KIRC, LUAD, LIHC, CESE, and GMB (Figure 6A). The immune interaction and mechanism between PTPN7 and infiltration derived by immune cells in pan-cancer was analyzed, PTPN7 was negatively correlated with tumor purity but positively correlated with multiple immune cells infiltration in most cancer types (Figure 6B). In addition, we also plotted the interaction between PTPN7 and PD-L1 & CTLA-4 expression. It could be observed that PTPN7 was positively correlated with PD-L1 and CTLA-4 expression across almost all cancer types (Figures 6C,D). The R and *p* values were displayed in Supplementary Table S3 and Supplementary Table S4. Collectively, these findings uncover that PTPN7 may play a vital role in the tumor immunity in the pan-cancer.

### 3.6 PTPN7 predicts the response to immunotherapy

**FIGURE 7:**
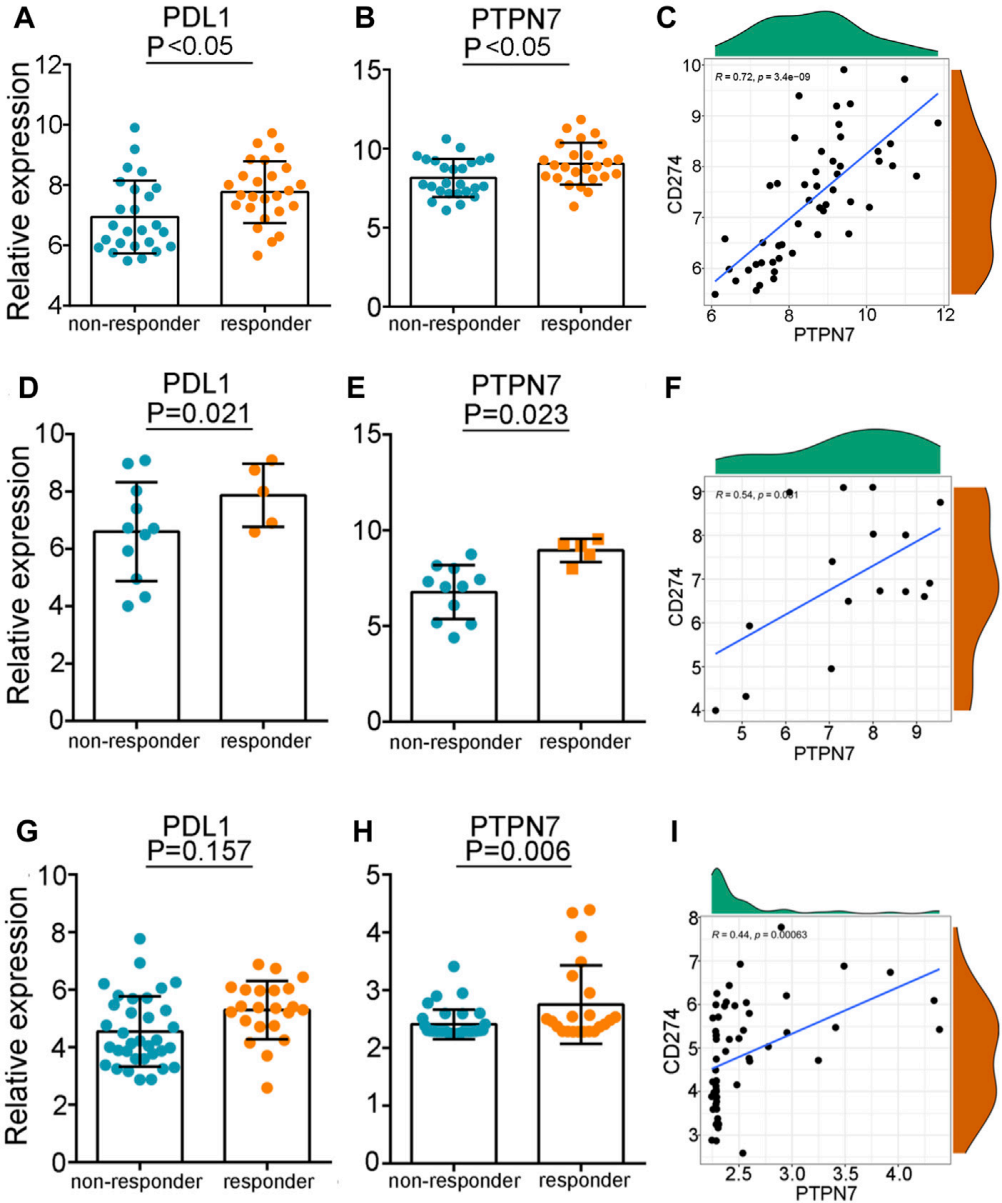
The predictive values of PTPN7 for immunotherapy. **(A,D,G)** Expression of PD-L1 in patients with different responses to immunotherapy in the three GEO cohorts. **(B,E,H)** Expression of PTPN7 in patients with different responses to immunotherapy in the three GEO cohorts. **(C,F,I)** Correlation between PD-L1 and PTPN7 in the three GEO cohorts.

Given the pan-cancer positive correlation between PTPN7 and immuno-factors, we speculated that PTPN7 could be an innovative biomarker to forecast the consequence to immunotherapy. In the GSE35640 dataset, PD-L1 and PTPN7 were highly expressed in patients with a better immunotherapeutic response (Figures 7D,E), and PTPN7 was positively correlated with PD-L1 expression (Figure 7H). What’s more, we found in the GSE126044 dataset, the expression of PD-L1 was not varied while PTPN7 was upregulated in patients with a better immunotherapeutic response (Figures 7G,H). The positive gradient momentum between PTPN7 and PD-L1 was also observed in the GSE126044 dataset (Figure 7I). Additionally, we compared PTPN7 with PD-1 in the three individual clinical cohort which PRJNA558949, GSE35640 and GSE126044 included. There has the similar diagnostic performance in the TNBC and early-stage lung cancer (Supplementary Figure S2A,B). Interestingly, we observe that PTPN7 is kind of PD-1’s substitute in the non-small cell lung cancer which performs almost equal contribution as PD-1 (Supplementary Figure S2C). Overall, PTPN7 can be a novel biomarker to predict the response to immunotherapy in cancer.

## 4 Discussion

PTPNs dominate tyrosine phosphorylation and vice versa which in the cell level of signal transmission with protein-tyrosine kinases, as members of the protein tyrosine phosphatases family (Tonks, 2006). There have been several studies focusing on the connections between specific PTPN members and various neoplasms (Chen et al., 2020; Cubas et al., 2020; Wang et al., 2021b). Interestingly, to date, no coverage has outlined how the PTPN family genes are linked to BrCa and the interaction between the PTPN family and pan-cancer. To reveal the relationship between PTPNs and BrCa and pan-cancer, we conducted a demonstration of PTPN7 as a biomarker in predicting the response of immunotherapy to diverse tumors that provide immuno-hot signals.

In this study, we used large-scale RNA-seq data to analyze PTPNs and experimentally verified the role of PTPNs in BrCa. We discovered that PTPN7 was adversely connected with tumor purity in BrCa tumor types, but positively correlated with numerous immune cell infiltration. The intracellular content level of PTPNs in BrCa found in public databases was inconsistent, but PTPN7 was overexpressed in tumor tissues compared to paired para-tumor tissues in the current cohort. As for the prognostic values of PTPNs in BrCa, high expression of PTPN7 was associated with a better prognosis. It has been established that PTPN7 plays a role in the control of T cell antigen receptor (TCR) signaling, which is considered to work by dephosphorylating the molecules connected to the MAP kinase pathway. (Petersen et al., 1999) (Gene ID: 5778) However, the functional role of PTPN7 in BrCa remains to be further studied.

Compared to other cancer types, Most of BrCa subtypes have traditionally been thought to be an immune-cold except (Xie et al., 2020); However, TNBC shows the greatest promise in the application of immunotherapy, and the lack of targeted therapeutic options makes it especially important to find new targets (Berger et al., 2021). This study reveals the association between the expression of PTPNs and the gene markers of multiple immunological checkpoints and immune invading cells were shown to be highly linked with the expression of PTPN7. The correlation between PTPN7 and these immune characteristics suggests that PTPN7 plays an important role in regulating the tumor immune microenvironment that indicates PTPN7 would be a promising biomarker for the immunotherapy sensitivity or the thermal and cold pointer vice versa.

The pan-cancer immunoassay was begun in 2017 (Lee et al., 2008; Charoentong et al., 2017). As the first immunoassay special biomarker monoclonal antibody RP215 had been revealed, the research has been put into a new level of the analysis which built several cohorts of gene family and annalistic tools, our study is aimed to provide a promising biomarker during PTPN family in order to enlarge the recognition of tumor regulation and genesis. What’s more, high TMB consistently selects for ICB treatment benefits. By whole-exome sequencing, the current predicted TMB thresholds proposed more than 190 non-synonymous somatic mutations which observed in lung, bladder, brain, and neck malignancies (WES) (Cai et al., 2021). In high TMB tumors treated with single-agent PD-(L)1 antibody, PD-L1 expression influences response to ICB; however, response to anti-CTLA4 or anti-PD-1/CTLA-4 combination therapy cloud not able to influence by PD-L1 expression (Huang et al., 2021). There are no well-established disease-specific TMB for predicting response in a variety of different cancers. Therefore, our study presents a novel biomarker that could be a pointer to predicting the response for certain cancer during immunotherapy. Last but not least, as the function of PTPN7 has been reported that this PTP was discovered to be involved in the control of TCR signaling, which is hypothesized to work by dephosphorylating molecules involved in the MAP kinase pathway (Inamdar et al., 2019). The phenomena of PTPN7 which overexpressed during the pan cancer genesis is prospective for in-depth research that may be helpful to recognized PTPN7 as a novel pan cancer biomarker.

## 5 Conclusion

In summary, this is the first comprehensive investigation of the relationship between PTPN family expression and clinical features in BrCa which represent a promising biomarker for immunotherapy prediction. Concretely, PTPN7 expression is associated with immuno-hot tumors in BrCa and multiple cancers, which could be a promising predictive biomarker for immunotherapy.

## Data Availability

The datasets presented in this study can be found in online repositories. The names of the repository/repositories and accession number(s) can be found below: https://www.jianguoyun.com/p/DbyItS0QwY7NChj_378EIAA.

## Abbreviations

TCGA, the Cancer Genome Atlas; RNA-seq, RNA-sequencing; GEO, Gene Expression Omnibus; PFS, progression-free survival; OS, overall survival; TME, tumor microenvironment; TIIC, tumor-infiltrating immune cell; IPS, immunophenoscore; TMA, tissue microarray; BrCa, breast cancer; TNBC, riple-negative breast cancer; HR, hazard ratio; KEGG, Kyoto Encyclopedia of Genes and Genomes; TIMER, tumor Immune Estimation Resource.
