# Supplementary material for: Comprehensive analysis of PTPN gene family revealing PTPN7 as a novel biomarker for immuno-hot tumors in breast cancer
Source: Front Genet. 2022 Sep 26;13:981603. doi: 10.3389/fgene.2022.981603 (PMC9548886; doi:10.3389/fgene.2022.981603)
Supplement: Supplementary file 1 [file Table1.DOCX]

**Supplementary Tables**

**Table S1. GO analysis of PTPN family based on the DAVID tool.**

| **Category** | **Term** | **Annotation** | **Count** | **FDR** |
| --- | --- | --- | --- | --- |
| BP | GO:0035335 | peptidyl-tyrosine dephosphorylation | 18 | <0.001 |
| BP | GO:0006470 | protein dephosphorylation | 13 | <0.001 |
| BP | GO:0050860 | negative regulation of T cell receptor signaling pathway | 3 | 0.009 |
| BP | GO:0060338 | regulation of type I interferon-mediated signaling pathway | 3 | 0.015 |
| BP | GO:0043407 | negative regulation of MAP kinase activity | 3 | 0.024 |
| CC | GO:0005737 | cytoplasm | 15 | <0.001 |
| CC | GO:0009898 | cytoplasmic side of plasma membrane | 4 | <0.001 |
| CC | GO:0005856 | cytoskeleton | 6 | <0.001 |
| CC | GO:0005634 | nucleus | 10 | 0.332 |
| CC | GO:0019898 | extrinsic component of membrane | 2 | 0.517 |
| MF | GO:0004725 | protein tyrosine phosphatase activity | 18 | <0.001 |
| MF | GO:0004726 | non-membrane spanning protein tyrosine phosphatase activity | 7 | <0.001 |
| MF | GO:0030971 | receptor tyrosine kinase binding | 4 | <0.001 |
| MF | GO:0001784 | phosphotyrosine binding | 3 | <0.001 |
| MF | GO:0005515 | protein binding | 17 | 0.002 |

**Table S2. GO analysis of PTPN7 in BrCa based on the Linked Omics tool.**

| **Category** | **Term** | **Annotation** | **Count** | **FDR** |
| --- | --- | --- | --- | --- |
| BP | GO:0071806 | protein transmembrane transport | 59 | 0.010 |
| BP | GO:0032633 | interleukin-4 production | 34 | 0.016 |
| BP | GO:0001773 | myeloid dendritic cell activation | 27 | 0.024 |
| BP | GO:0050690 | regulation of defense response to virus by virus | 29 | 0.027 |
| BP | GO:0045730 | respiratory burst | 27 | 0.038 |
| CC | GO:0001772 | immunological synapse | 32 | 0.012 |
| CC | GO:0042629 | mast cell granule | 21 | 0.013 |
| CC | GO:0042611 | MHC protein complex | 19 | 0.016 |
| CC | GO:0071782 | endoplasmic reticulum tubular network | 18 | 0.018 |
| CC | GO:0001891 | phagocytic cup | 21 | 0.024 |
| MF | GO:0016502 | nucleotide receptor activity | 22 | 0.009 |
| MF | GO:0003823 | antigen binding | 52 | 0.009 |
| MF | GO:0015026 | coreceptor activity | 39 | 0.012 |
| MF | GO:0042287 | MHC protein binding | 24 | 0.019 |
| MF | GO:0038187 | pattern recognition receptor activity | 20 | 0.020 |

**Table S3. Correlation analysis between PTPN7 and PD-L1 in pan-cancer.**

| **Cancer type** | **R value** | **P value** |
| --- | --- | --- |
| ACC | 0.413 | <0.001 |
| BLCA | 0.643 | <0.001 |
| BRCA | 0.712 | <0.001 |
| CESC | 0.441 | <0.001 |
| CHOL | 0.639 | <0.001 |
| COAD | 0.613 | <0.001 |
| DLBC | 0.499 | <0.001 |
| ESCA | 0.396 | <0.001 |
| GBM | 0.412 | <0.001 |
| HNSC | 0.572 | <0.001 |
| KICH | 0.155 | 0.214 |
| KIRC | 0.410 | <0.001 |
| KIRP | 0.277 | <0.001 |
| LGG | 0.403 | <0.001 |
| LIHC | 0.530 | <0.001 |
| LUAD | 0.623 | <0.001 |
| LUSC | 0.430 | <0.001 |
| MESO | 0.478 | <0.001 |
| OV | 0.618 | <0.001 |
| PAAD | 0.532 | <0.001 |
| PCPG | 0.295 | <0.001 |
| PRAD | 0.680 | <0.001 |
| READ | 0.574 | <0.001 |
| SARC | 0.415 | <0.001 |
| SKCM | 0.741 | <0.001 |
| STAD | 0.552 | <0.001 |
| TGCT | 0.791 | <0.001 |
| THCA | 0.578 | <0.001 |
| THYM | 0.064 | 0.487 |
| UCEC | 0.458 | <0.001 |
| UCS | 0.588 | <0.001 |
| UVM | 0.516 | <0.001 |

**Table S4. Correlation analysis between PTPN7 and CTLA-4 in pan-cancer.**

| Cancer type | R value | P value |
| --- | --- | --- |
| ACC | 0.157 | 0.166 |
| BLCA | 0.896 | <0.001 |
| BRCA | 0.841 | <0.001 |
| CESC | 0.800 | <0.001 |
| CHOL | 0.748 | <0.001 |
| COAD | 0.637 | <0.001 |
| DLBC | 0.288 | 0.047 |
| ESCA | 0.738 | <0.001 |
| GBM | 0.468 | <0.001 |
| HNSC | 0.716 | <0.001 |
| KICH | 0.739 | <0.001 |
| KIRC | 0.784 | <0.001 |
| KIRP | 0.647 | <0.001 |
| LGG | 0.369 | <0.001 |
| LIHC | 0.864 | <0.001 |
| LUAD | 0.812 | <0.001 |
| LUSC | 0.782 | <0.001 |
| MESO | 0.670 | <0.001 |
| OV | 0.724 | <0.001 |
| PAAD | 0.816 | <0.001 |
| PCPG | 0.686 | <0.001 |
| PRAD | 0.756 | <0.001 |
| READ | 0.502 | <0.001 |
| SARC | 0.738 | <0.001 |
| SKCM | 0.593 | <0.001 |
| STAD | 0.674 | <0.001 |
| TGCT | 0.899 | <0.001 |
| THCA | 0.909 | <0.001 |
| THYM | -0.318 | <0.001 |
| UCEC | 0.736 | <0.001 |
| UCS | 0.563 | <0.001 |
| UVM | 0.729 | <0.001 |
