# Supplementary material for: Comprehensive analysis of PTPN gene family revealing PTPN7 as a novel biomarker for immuno-hot tumors in breast cancer
Source: Front Genet. 2022 Sep 26;13:981603. doi: 10.3389/fgene.2022.981603 (PMC9548886; doi:10.3389/fgene.2022.981603)
Supplement: Supplementary file 3 [file Table3.DOCX]

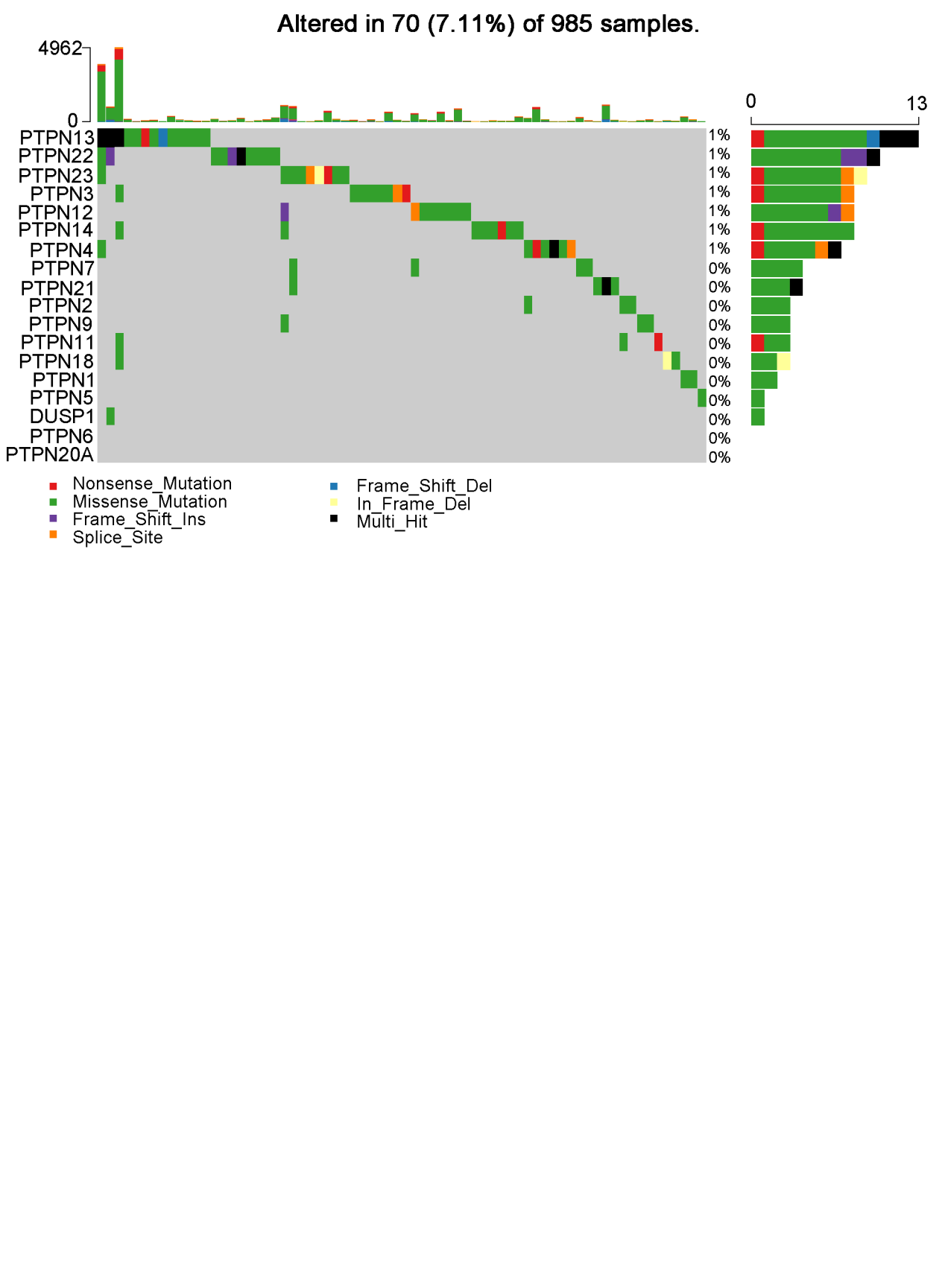


**Figure S1: Mutational profiles of PTPNs in the TCGA cohort.**


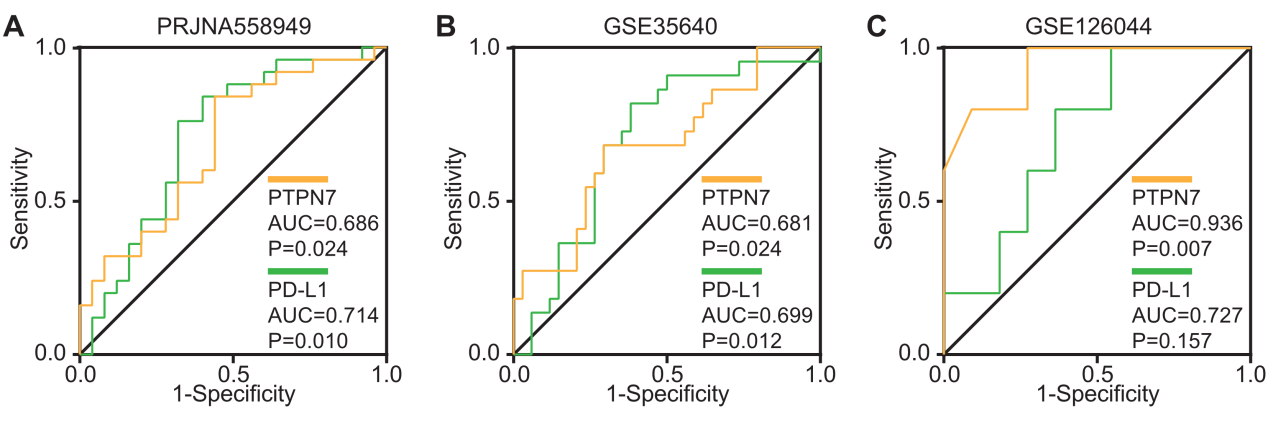
**Figure S2: Comparison of PTPN7 and PDL1 in clinical efficacy and prognostic value**
