# Supplementary material for: Comprehensive analysis of PTPN gene family revealing PTPN7 as a novel biomarker for immuno-hot tumors in breast cancer
Source: Front Genet. 2022 Sep 26;13:981603. doi: 10.3389/fgene.2022.981603 (PMC9548886; doi:10.3389/fgene.2022.981603)
Supplement: Supplementary file 2 [file Table2.DOCX]

Dear Editor,

I would like to submit to *Frontiers in Genetics* our original manuscript entitled “**Comprehensive analysis of PTPN gene family revealing PTPN7 as a novel biomarker for immuno-hot tumors in breast cancer**” as a research article. All authors have approved this submission. None of the materials in this manuscript has been published or is under consideration elsewhere. None of the authors has a conflict of financial interest.

The non-receptor protein tyrosine phosphatase (PTPN) gene family has been considered to be involved in the oncogenesis and development of multiple cancers. However, its prognostic utility and immunological relevance in breast cancer (BrCa) have not been clarified. A transcriptional level interpretation of the expressions and prognostic values was analyzed using the data from The Cancer Genome Atlas (TCGA) cohort. In addition, GO analysis pinpoints the functional enrichment of PTPNs. Moreover, the immune correlations of PTPN7 in BrCa and pan-cancer were further investigated based on the TCGA cohort and were testified using the in-house and the Gene Expression Omnibus (GEO) cohorts. For systematic analysis of the PTPN family, we found that the expression levels of PTPN1, PTPN6, PTPN7, PTPN18, PTPN20, and PTPN22 was promoted in tumor tissues while comparing with paraneoplastic tissues during our study. We further investigated their functions and protein-protein interactions (PPI), and these results strongly suggested that PTPN family was associated with protein dephosphorylation. Next, we performed an immunological relevance analysis and found that PTPN7 was correlated with immune infiltration, suggesting a stronger association of PTPN7 with immuno-hot tumors in BrCa. In addition, results from the in-house cohort confirmed the positive correlation between PTPN7 and PD-L1. The pan-cancer analysis revealed that PTPN7 was related to PD-L1 and CTLA-4 expression in almost all cancer types. Finally, the predictive value of PTPN7 for immunotherapy was significant in two independent GEO cohorts. In conclusion, this is the first extensive research on the correlation between PTPN family expression and immune characterization in BrCa. As results, PTPN7 expression is associated with immuno-hot tumors and could be a promising predictive biomarker for immunotherapy in not only BrCa but multiple cancers.

Thank you for your time and we look forward to your favorable consideration.

Sincerely yours!

Xinyuan Zhao, Ph.D.

Department of Occupational Medicine and Environmental Toxicology,

School of Public Health, Nangtong University, Nantong 226019, China

Phone: +86-0513-85012912

Email: [zhaoxinyuan@ntu.edu.cn](mailto:zhaoxinyuan@ntu.edu.cn)

As the picture above, our raw data, analysis codes and raw pictures including IHC raw images are contained in jianguoyun link.
